# Overweight, Obesity, and Age Are the Main Determinants of Cardiovascular Risk Aggregation in the Current Mexican Population: The FRIMEX III Study

**DOI:** 10.3390/jcm13082248

**Published:** 2024-04-12

**Authors:** Eduardo Meaney, Enrique Pérez-Robles, Miguel Ortiz-Flores, Guillermo Perez-Ishiwara, Alejandra Meaney, Levy Munguía, Gisele Roman, Nayelli Nájera, Guillermo Ceballos

**Affiliations:** 1 Laboratorio de Investigación Cardiometabólica Integral, Sección de Estudios de Posgrado e Investigación, Escuela Superior de Medicina, Instituto Politécnico Nacional, Mexico City 11340, Mexicoeperezrobles@gmail.com (E.P.-R.); maortizfl@ipn.mx (M.O.-F.); 2 Escuela Nacional de Medicina y Homeopatía, Instituto Politécnico Nacional, Mexico City 07320, Mexico; dperez@ipn.mx; 3 Cardiovascular Unit, Hospital Regional “1° de Octubre”, Instituto de Seguridad Social y Servicios para los Trabajadores del Estado, Mexico City 07760, Mexico; 4 Dirección Normativa de Salud, Instituto de Seguridad y Servicios Sociales para los Trabajadores del Estado, Mexico City 06030, Mexico; 5 Coalición por el Corazón de México, Mexico City 01090, Mexico

**Keywords:** total cholesterol, glycemia, systolic blood pressure, body mass index, multiple linear regression

## Abstract

**Background**: The Mexican population exhibits several cardiovascular risk factors (CVRF) including high blood pressure (HBP), dysglycemia, dyslipidemia, overweight, and obesity. This study is an extensive observation of the most important CVFRs in six of the most populated cities in Mexico. **Methods**: In a cohort of 297,370 participants (54% female, mean age 43 ± 12.6 years), anthropometric (body mass index (BMI)), metabolic (glycemia and total cholesterol (TC)), and blood pressure (BP) data were obtained. **Results**: From age 40, 40% and 30% of the cohort’s participants were overweight or obese, respectively. HBP was found in 27% of participants. However, only 8% of all hypertensive patients were controlled. Fifty percent of the subjects 50 years and older were hypercholesterolemic. Glycemia had a constant linear relation with age. BMI had a linear correlation with SBP, glycemia, and TC, with elevated coefficients in all cases and genders. The β1 coefficient for BMI was more significant in all equations than the other β, indicating that it greatly influences the other CVRFs. **Conclusions**: TC, glycemia, and SBP, the most critical atherogenic factors, are directly related to BMI.

## 1. Introduction

Contemporary cardiovascular (CV) and cardiometabolic medicines are strongly oriented to fundamental, primary, secondary, and tertiary phases of prevention, basing the prophylaxis and treatment of CV and cardiometabolic diseases on therapeutic modifications of lifestyle, altogether with proven pharmacological and interventional therapies [1,2,3]. In this context, determining and characterizing the risk profiles of a given population are essential for developing proper public policies and therapeutic and preventive guidelines to mitigate the prevalence and lethality of the epidemic flagella that assail contemporary society.

Cardiac diseases (76.3% are ischemic heart disease) and diabetes mellitus (DM) are the leading causes of total mortality in Mexico, provoking 200,535 deaths in 2022 [4]. Several governmental and academic studies have revealed the high-risk profile affecting the contemporary Mexican population [5,6,7,8,9,10]. The FRIMEX study [11], an RF survey in six of the most populated cities in Mexico, including more than 140,000 persons of both genders, with an average age of 44 ± 13 years, revealed a population with very high cardiovascular risk, where overweight or obesity (O/O) affected 71.9% of participants, in straight correlation with high blood pressure (HBP), hypercholesterolemia, and elevated glucose serum concentrations. This study and many others indicate that Mexico’s leading CV and cardiometabolic risk factor (RF) is O/O, as in many other countries and regions worldwide. However, despite the evident tragic current Mexican epidemiologic panorama, informative and preventive programs have not been established nor have solid public policies that mitigate the prevalence of O/O and their consequences been implemented.

Obesity is a chronic, heterogeneous, relapsing, and progressing structural disease [12] due to the loss of balance between caloric intake and energy expenditure. It is characterized by the excessive accumulation and abnormal distribution of body fat. It is frequently associated with structural and functional alterations of adipocytes as well as hypertrophy, hyperplasia, ischemia, necrosis, apoptosis, and autophagy of fatty tissue. At the same time, it is often accompanied by resistance to insulin and secondary hyperinsulinism, inflammation, oxidative stress, and endothelial dysfunction. The last triad leads to the development of morbid conditions that affect multiple organs and systems [13]. According to the World Health Organization (WHO), obesity is classified, using the body mass index (BMI, kg/m^2^), into the following three categories: class 1 (BMI of 30 to <35), class 2 (BMI of 35 to <40), and class 3 (BMI of 40 or higher) [14,15]. In many studies, a strong linear relationship between the degree of obesity and the occurrence of HBP, stroke, and myocardial infarction has been demonstrated [16].

Numerous mechanisms link O/O to the development of multiple cardiovascular diseases, mainly atherosclerotic cardiovascular diseases (ASCVD), but also to heart failure, chronic kidney disease, and obstructive sleep apnea, among many others. Amid several complex mechanisms stand out the already mentioned vascular pathogenic triad. This includes the inflamed ectopic pericardial fat tissue; the binomial insulin resistance/hyperinsulinism; obesity-related comorbidities such as high blood pressure (HBP), dyslipidemia, and diabetes; and systemic inflammation and adverse cytokines, as well as a prothrombotic milieu and the overexpression of renin–angiotensin–aldosterone axis, among others [17,18].

The present report does not aim to show our population’s already known pattern of high risk but to highlight the importance of the close interrelationship among the most crucial CVRFs. Moreover, the analysis of this large cohort should show the hierarchical importance of the different CVRFs studied in this survey to profile the risk pattern of the contemporary Mexican population. Likewise, this complex tangle of CV disease-promoting agents imposes therapeutic and preventive strategies that must consider these interrelationships to establish a holistic treatment, better reducing the severity of all CVRFs and metabolic risks.

## 2. Materials and Methods

The methodology of this study has already been published [11]. The study was conducted following the guidance of Good Clinical Practices [19], the norms from the Declaration of Helsinki, [20] and the Mexican Federal regulations established in the General Law of Health [21]. Mobile units, served by trained health personnel, were placed in commercial malls or civic centers and squares in six of the most populated cities in the country, some located in Central Mexico (the capital Mexico City, Puebla, and León), others in the central western geographic area (Guadalajara), and two in the northernmost regions (Monterrey and Tijuana). The participants, aged 18 years or older, of any gender, were recruited by invitation. The design of this survey allowed the recruitment of any adult who accepted the invitation. Therefore, there were no inclusion criteria. The cohort comprised a non-probabilistic sample of 300,000 participants of both genders (recruited in the last 10 years). Body mass index (BMI, kg/m^2^) was obtained in all subjects through weight and height and was classified, according to the WHO, [15] as underweight (BMI < 18.5), healthy weight (BMI 18.5–24.9), overweight (BMI 25.0–29.9), and obesity (BMI ≥ 30.0). Blood pressure (BP) was measured with calibrated mercurial sphygmomanometers following standard recommendations [22]. HBP was considered when systolic blood pressure (SBP) was ≥140 mm Hg associated or not to a diastolic blood pressure (DBP) ≥90 mm Hg, in concordance with the cut-offs accepted by the official Mexican norm on hypertension [23]. The proportions known as the “law of the halves” [24] were estimated from the data collected in the survey which includes the rates of awareness, treatment, and control (treatment type was not recorded). Glucose was measured in capillary blood with a “dry chemistry” apparatus (Accutrend, Roche Diagnostics, Basel, Switzerland) and participants fasted for at least five hours. Dysglycemia was classified as fasting plasma glucose (FPG) between 100 and 125 mg/dL and overt DM with ciphers of ≥126 mg/dL or ≥200 mg/dL, regardless of fasting time, following recommendations of the American Diabetes Association [25]. Total cholesterol (TC) was measured with the same technique. Its concentrations, according to the Adult Treatment Panel III (ATP III), [26] were classified as adequate when TC was <200 mg/dL, borderline hypercholesterolemia when it was between 200 and 239 mg/dL, and definitive hypercholesterolemia with TC ≥ 240 mg/dL (due to logistic and economic restrictions, triglycerides, HDL, or LDL concentrations were not obtained). Finally, smoking status was established as regular consumers if the participants had smoked any amount of tobacco habitually within the last trimester or a longer time and non-smokers if they had never smoked or only occasionally. However, in this first analysis, we omitted tobacco’s contribution to CV risk because the number of cigarettes consumed was not registered.

### Statistics

Data were presented as mean ± SD; the analysis included year-by-year (20–70 years of age) changes in risk marker levels, an analysis of age-related changes by gender with linear or quadratic correlation between variables when applied. A multilinear regression analysis was performed to generate equations exploring the influence of each variable on body mass index, systolic blood pressure, and cholesterol. This study was conducted using Prism software, 10.1.1 version (www.graphpad.com). Statistical significance was considered when *p* < 0.05.

## 3. Results

Of the 300,000 participants recruited, those with incomplete data or records showing markedly out-of-range values were excluded, leaving just 297,370 for analysis. This small number, representing 0.87% of all surveyed subjects, cannot influence the analysis results. Most participants (29%) were from Mexico City, while in the rest of the cities, the proportion between them was very similar, between 11% and 15%. Regarding gender, 54% were female, and 46% were male. The mean age was 43 ± 12.6 years (men were 41 ± 11.7 years and women were 44 ± 12.7 years), the mode was 32.3 years, and the 31–40 decade was the most frequent. Concerning educational status, 54.5% of the cohort’s participants had elementary education, 21.1% had middle-high or technical subprofessional levels, and 24.4% had college or higher degrees. Considering genders, women obtained 61.5%, 19%, and 19.5% of the three academic mentioned categories, while men obtained 46.2%, 23.4%, and 30.3%, respectively. Table 1 shows the cohort’s anthropometric, metabolic, and BP data, as well as the totals and those corresponding to each gender. Although men were more overweight than women, no significant differences were observed between genders in the metabolic or BP data.

**Figure 1:**
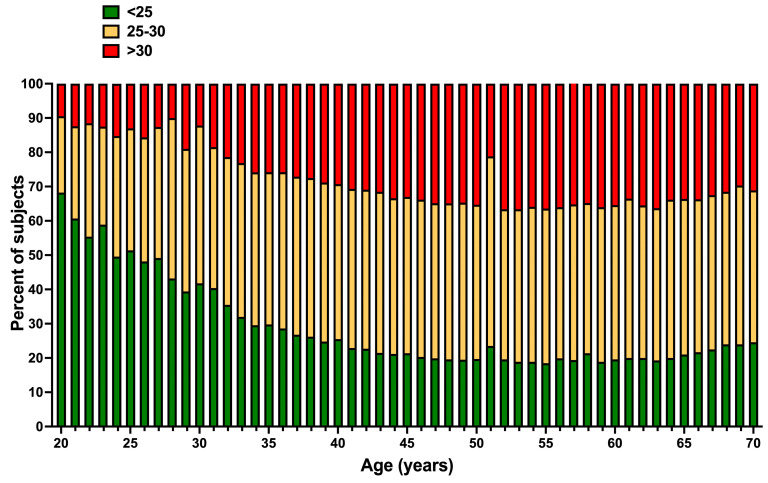
Percentage of normal body mass index (green), overweight (yellow), and obesity (red) by age. An apparent age-related decrease in the percentage of subjects having a normal BMI (<25) was found. An age-related increase in the percentage of subjects with overweight or obesity was also seen.

Figure 1 reveals that a healthy BMI, starting at age 20, decreases, giving rise to a progressively higher frequency of overweight and obesity (O-O). From the age of 40, O/O affects 75–80% of the study population (overweight was observed in about 40% of the participants and obesity in 30%).

**Figure 2:**
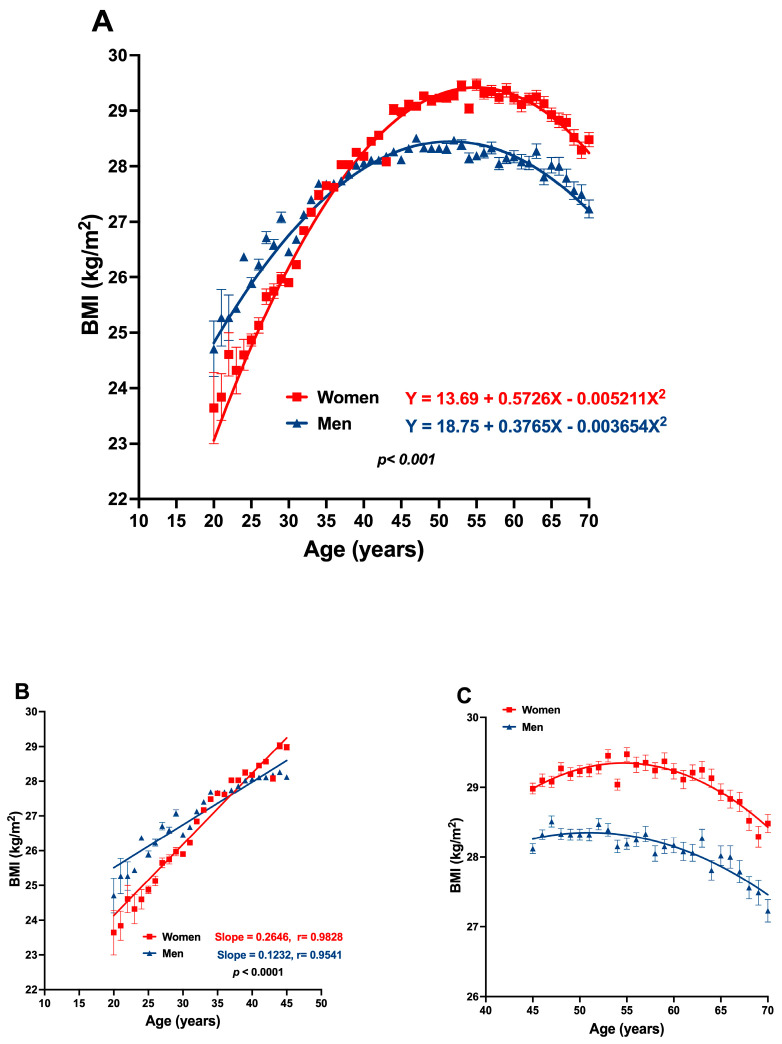
(**A**) Differential age-related BMI changes between women and men; a second-order behavior is appreciated. (**B**) A linear relation between age and BMI is found in the range of 20 to 45 years in both genders. (**C**) A plateau or decreased BMI was found as age advanced, from 45 to 70 years. Data are presented as mean ± SD.

In Figure 2, the relationship between BMI and age is shown. A straight linear relation exists between both variables from 20 to 40–45 years (with high linear correlation coefficients; r = 0.98 and 0.95 in women and men, respectively), from which the line horizontalizes, and, when reaching 60 or more years, it curves downwards. Men are more overweight than women up to 35 years of age, but from that age to 40, the BMI is similar in both genders. From age 45 years and older, women have a greater body mass than men.

**Figure 3:**
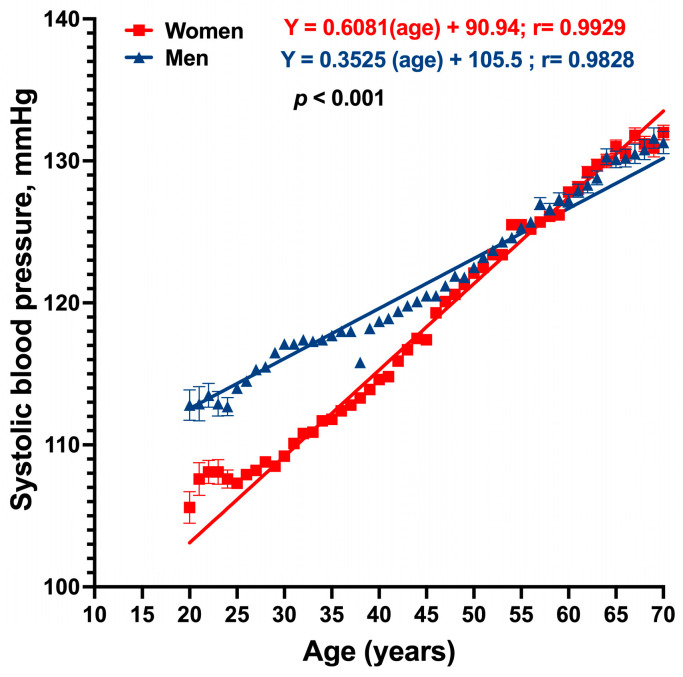
Mean and standard deviation are used to present data on age-related increases in systolic blood pressure.

On the contrary, Figure 3 reveals that systolic blood pressure (SBP) rises directly and constantly through all the age stages, with the slope being more significant in women than men. As expected, women showed lower SBP levels until 50–55 years old; there is no difference between genders after that age. The slopes (0.6081 and 0.3525 for women and men, respectively) suggested a higher increase in SBP in women over time.

**Figure 4:**
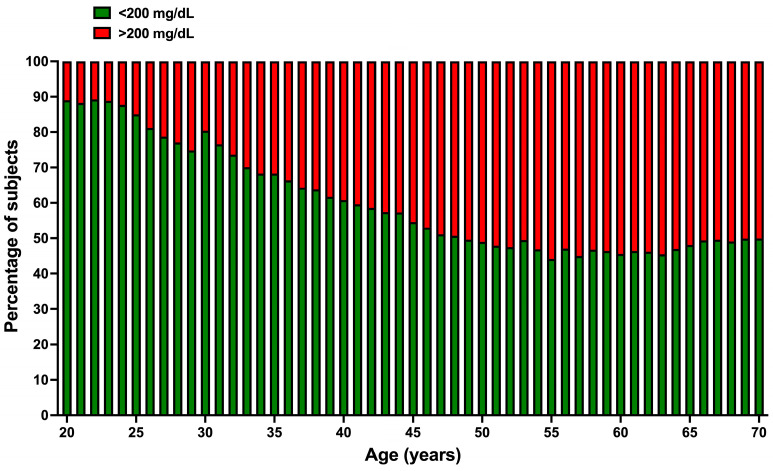
Percentage of normal cholesterol levels (green) and hypercholesterolemia (red) by age. An apparent age-related decrease in the percentage of subjects having normal cholesterol (<200) was found. An age-related increase in the percentage of subjects with hypercholesterolemia was also seen.

Figure 4 shows how the concentration of TC changes across age groups. Only 10 to 15% of participants had hypercholesterolemia at younger ages; however, from age 26, the proportion of subjects with hypercholesterolemia (CT greater than 200 mg/dL) increased progressively until age 50, when half of the study population was hypercholesterolemic.

**Figure 5:**
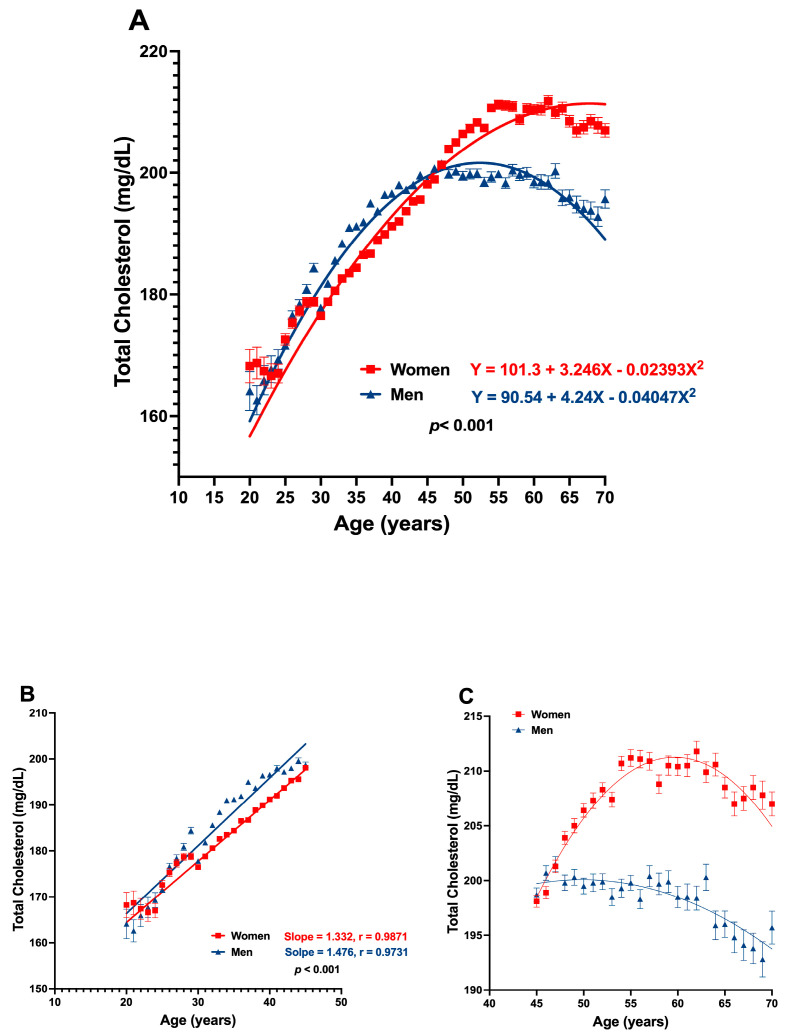
(**A**) Differential age-related cholesterol levels change between women and men; a second-order behavior is appreciated. (**B**) A linear relation between age and cholesterol was found in the range of 20 to 45 years in both genders. (**C**) A plateau or decreased cholesterol levels was found as age advanced, from 45 to 70 years. Data are presented as mean ± SD.

Figure 5 shows the same peculiarity between CT and age observed with the BMI. There is a positive and linear relationship between cholesterol and age up to 45 years, followed by a plateau, and, from 65 years, the correlation line is curved downwards. Women had higher values of CT than men, but the fall of this variable is more pronounced in men.

**Figure 6:**
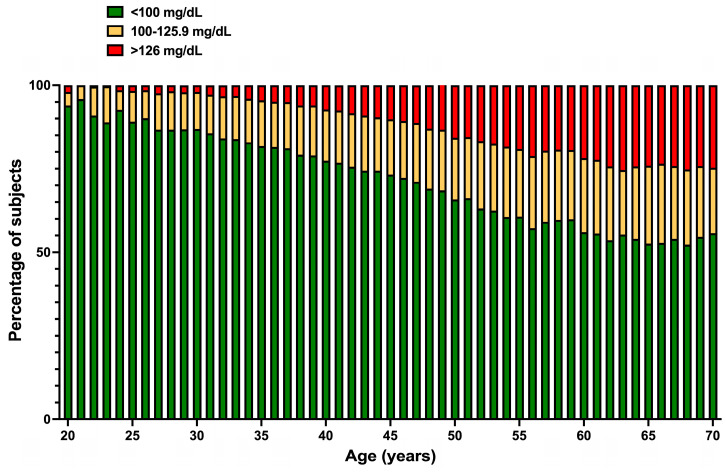
Percentage of normal glucose (green), hyperglycemia (yellow), and DM (red) glucose levels by age. An apparent age-related decrease in the percentage of subjects having normal glycemia (<100 mg/dL) was found. An age-related increase in the percentage of subjects with hyperglycemia and DM was also seen.

Interestingly, Figure 6 shows that glycemia is normal in 94% of youngsters at 20. From this age, the proportions of pre-diabetic dysglycemia and diabetes increase until a maximum at age 50, where the combination of both comprises 40% of the study’s population. From 60 years of age and older, around 30% of subjects have blood glucose levels greater than 126 mg/dL.

**Figure 7:**
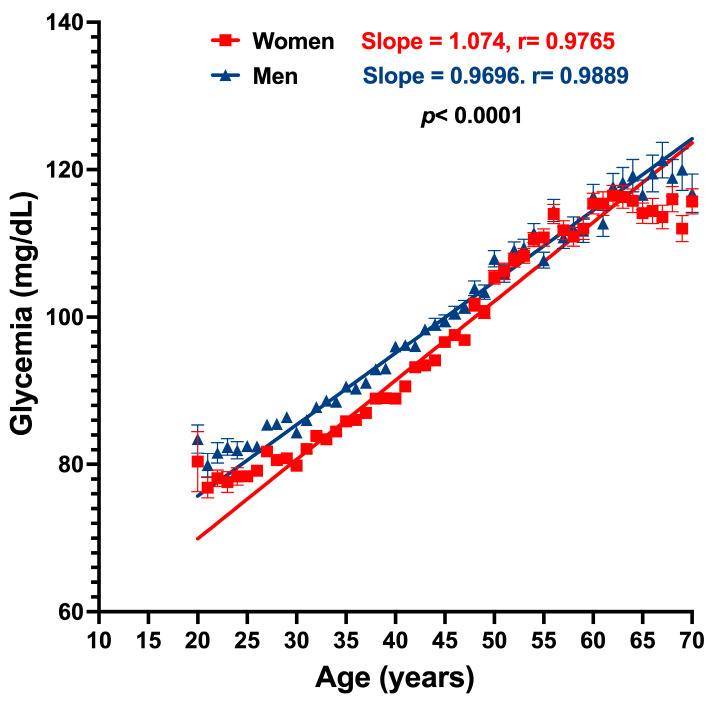
Differential age-related glycemia levels change between women and men; a linear behavior is appreciated in both genders (r ≥ 0.97). Data are presented as mean ± SD.

Figure 7 reveals that, contrary to what happens with BMI and TC, the correlation between glycemia and age is straightly linear (with very high correlation coefficients) from youth to old age.

**Figure 8:**
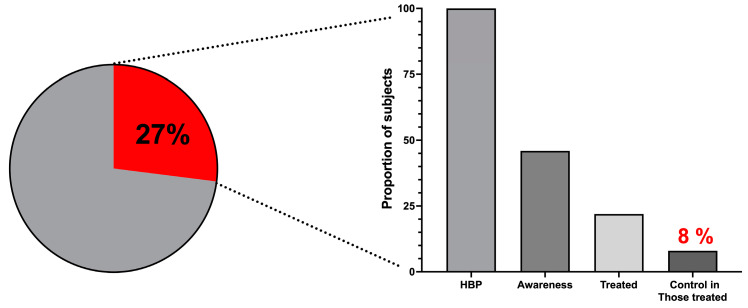
Of the entire cohort universe, 27% (the segment in red) had HBP. The rates of awareness, treatment, and hypertension control are displayed. This represents the “law of the halves” in the hypertensive study’s population.

On the other hand, Figure 8 shows the proportions of awareness, treatment, and control of the hypertensive population. Of the total cohort universe, 27% of the participants were hypertensives. Less than half were aware of their diagnosis (46.5%). About one-third (36%) of those treated had controlled BP, but the overall proportion of controlled hypertensive subjects was only 8%.

**Figure 9:**
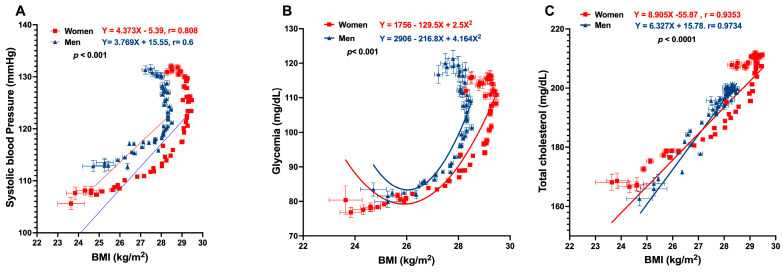
(**A**) shows a linear correlation between SBP and body mass up to a BMI of 28–29 kg/m^2^, from which the linear correlation is lost, and the line curves back and down; this behavior is similar in both genders. (**B**) A more complex relationship between BMI and serum glucose is shown. Even though an initial direct correlation was evident up to a BMI of 28–29 kg/m^2^, the correlation line also curves back and down later. In this case, the correlation coefficients are less striking. (**C**) Shows a more consistent linear relationship between TC and BMI, with a correlation coefficient greater than 0.9 in both genders.

The analysis of the relationship between BMI and SBP, glycemia, and TC is shown in Figure 9. A straight linear correlation between SBP and body mass index was found; however, the linear correlation was lost after a BMI of 28 kg/m^2^ when the line curves back and down. The correlation coefficients for women and men are relatively high (0.80 and 0.62, respectively). A more complex relationship between BMI and serum glucose was found. Even though an initial direct correlation was evident up to a BMI of 28–29 kg/m^2^, the correlation line also curves back and down later. In this case, the correlation coefficients are less striking (0.68 and 0.46 in women and men). On the other hand, there was a more consistent linear relationship between BMI and TC, with a correlation coefficient greater than 0.9 in both genders.

Due to these striking behaviors, we performed a multiple regression analysis. We used multiple regression analyses with the obtained variables to search for a model equation with the highest influence over the other variables. The following approaches were used: (1) the influence of BMI, age, and glycemia in the cholesterol levels; (2) the influence of BMI, cholesterol, and glycemia in SBP; and (3) the influence of age, cholesterol, and glycemia in BMI. The generated equations for both genders are described in Table 2.

## 4. Discussion

The results reported here highlight the importance of risk aggregation, which sums the individual components of a set of risks, signals their interrelation, and suggests that overweight/obesity (BMI) is the most relevant risk factor in this population.

Public health policies entail all the modern state’s actions to attain some general health goals of social and medical interest applicable in the short, medium, and long range [27,28]. The solidity and transcendence of a public policy depend, on the one hand, on its scientific and epidemiological foundation and, on the other hand, on how it is presented to the community so that the majority of the social segments involved accept it well.

In Mexico, the O/O has reached epidemic proportions and is well documented by the national surveys called ENSANUTs [29,30,31]. In ENSANUT 2022, O/O was found in 75.2% of the surveyed adult population, in 37.3% of school children, and in 41.1% of teenagers [29,31]. This first epidemic surge supports the secondary waves of DM2 and coronary syndromes [32,33]. Therefore, a pending duty requiring urgent fulfillment is the task of reducing the incidence and prevalence of O/O against powerful economic and political interests and the prejudices and erroneous decisions in the way of life of a society influenced by medical misinformation, marketing propaganda, and unawareness.

In this sense, preventive measures must be based on the facts that link obesity to cardiovascular disease, mainly to ischemic heart disease, the most important epidemiological scourge of contemporary Mexico. Since dyslipidemia (particularly, atherogenic dyslipidemia), HBP, binomial insulin resistance/hyperinsulinism, DM, and inflammation, some of the most important agents promoting atherosclerosis, depend linearly on the grade of obesity, weight loss alone would have a favorable preventive effect. This means that a single preventative therapeutic intervention will have multiple beneficial consequences, improving the overall aggregate risk.

The data in the present analysis indicate that O/O and age are the more influential independent determinants of other analyzed risk factors. The data signal that BMI, glycemia, TC concentration, and BP levels increase from youth to middle-aged adult life (50 or 55 years).

The so-called “law of the halves” is a valuable tool to measure a community’s medical and hygienic education level, the fortress of the political policies addressed to the prevention and clinical management of HBP, and the quality of medical care. In developed countries, the principle has evolved to a level called the “law of the thirds”, which means that a third of hypertensive patients are unaware of having the disease, another third is treated and controlled, and another third is uncontrolled even if they are treated [28,34]. In the sample analyzed in this work, less than half of the HBP patients were aware, less than half of those who were aware were treated, and a little more than a third of those treated were controlled, with an overall meager 8% of controlled concerning all the universe of hypertensive patients. As the survey was designed, no type of treatment with antihypertensive and lipid-lowering drugs was inquired about. However, at the time data collection was performed, the most frequent treatment in the institutional family care clinics for hypertension was a relatively selective beta-blocker (metoprolol), an ACEI such as captopril or enalapril, sometimes a calcium channel blocker such as amlodipine, and more frequently an ARB such as losartan. Most general physicians used the lowest possible doses of monotherapy, and combined therapy was rarely prescribed.

On the other hand, the only statin prescribed at that time in the public sector was pravastatin, at a modest dose of 10 mg per day. When hypertriglyceridemia was detected, bezafibrate was used. In the private sector, general practitioners who see the bulk of the population with modest resources, drugs, and therapeutic approaches are like those employed in health institutions. In contrast, medical specialists generally serving the middle and upper middle class utilize a wide range of antihypertensives and lipid-lowering agents influenced by American and European guidelines.

The data shown herein agree with the common Mexican epidemiological picture, where combining multiple risk factors constitutes an important atherogenic and dysmetabolic network. All these aggregated CVRFs are biologically and epidemiologically intertwined and influence each other. The population modifies a healthy profile at relatively young ages and shows a growing incidence of O/O, hypercholesterolemia, rising BP, and dysglycemia. When multiple regression equations were employed, it was evident that BMI significantly influences BP and TC. The value of the β coefficient for BMI (β1) in the TC regression equation for SBP was 3.963 in women and still more important in men. This coefficient signals to what extent the dependent variable increases when the independent variable becomes more prominent, while the other explanatory variables remain unchanged. BMI had less influence on SBP, which the regression equation showed β1 coefficients of 0.4703 in women and 1.593 in men. The β coefficient for BMI was more significant in all equations than the other β. Of all parameters, TC showed a more consistent linear correlation with BMI, with a regression coefficient of 0.9734 in men and slightly less in women of 0.9353. At the highest part of the regression line in women, it curves backwards, signaling that with a maximum BMI, TC no longer rises. In contrast, in men, both variables have a constant positive correlation.

The linearity of most correlations is affected by a phenomenon we already described above. Comparing BMI and TC against age, the linearity is lost once the age of 45 is reached. It is known that TC and LDL-c (cholesterol of the low-density lipoproteins) reach an equilibrium up to 65 years, and then their concentration decreases in both genders [35]. This phenomenon has many possible single or associated explanations that need to be clarified, such as weight loss, concomitant inflammatory chronic diseases, senescent less cholesterol intestinal absorption, and administration of lipid-lowering drugs, among others [36]. In the same context, it has been proved that BMI is a less reliable index of corpulence in the elderly, because, at this stage of life, some changes occur, like less appetite, sarcopenia, osteopenia, loss of height, and concomitant debilitating diseases, among others [37].

On the other hand, we observed the rise of SBP through the age scale explained by senescent changes in elastic arteries, like extracellular matrix dystrophy, calcification, and the development of atherosclerotic plaques [38].

Interestingly, a bizarre curving is observed in both genders in the multiple regression lines of glycemia and BP. In contrast to the behavior of the TC data, the regression line curves backwards and downwards. A theoretical explanation could be that in some aged subjects, there is a loss of weight explained for a handful of reasons, such as sarcopenia, uncontrolled diabetes, better medical care, patient awareness, intercurrent debilitating diseases, and the like. It is known that losing weight improves insulin sensitivity and intra- and extra-renal compression, which are significant mechanisms in dysglycemia and hypertension [39,40].

However, linearity is imperfect in SBP and glycemia multiple regression lines. Still, the correlation coefficient is high in SBP in both women (r = 0.808) and men (r = 0.625) and is less remarkable, but it is significant in glycemia (r = 0.68 in women and 0.46 in men).

## 5. Conclusions

Obesity (BMI) and age are the more prominent CVRFs, influencing the expression of each other, whose magnitude is aggravated by aging and adiposity. Cholesterol is the most important atherogenic CVRF. The data presented here demonstrate that its serum value is directly related to BMI. From the point of view of public policies, preventive programs directed at lessening coronary atherosclerotic disease must focus primarily on O/O. To this day, considerable efforts are devoted to reducing atherogenic lipids with statins, arterial hypertension with various antihypertensive drugs, and dysglycemia with a set of antidiabetic medications. However, health authorities and individual physicians pay less attention to the primal, basic condition of O/O. Therefore, it is essential to universalize in health institutions, in the therapeutic and preventive practices of health providers, and in the social imagination the importance of a healthy diet and the frequent practice of physical exercise, starting from childhood, to prevent the surge of the entire cluster of diseases and cardiometabolic syndromes, which are nowadays the leading public health threats. Additionally, the dietary and exercise approach reduces, primarily, cardiometabolic and CV risks and improves patients’ prognosis and quality of life in secondary prevention. Altogether, the therapeutic modifications of lifestyle, with pharmacological and bariatric treatments and procedures, can benefit society and individual patients [13,35,41]. We need, in addition, a national crusade, powerful and persistent, against obesity to influence the social imagination positively. Only in this way will we subdue or at least diminish the scourge of secondary epidemics of diabetes and ASCVD.

Despite its social lags, Mexico, now considered a middle-income country, is not far from achieving the status of an industrialized nation. But from now on, we suffer from what we have called the “Mexican paradox”, meaning that it is a slightly prosperous nation but with an impoverished population affected by the burden of disease typical of developed countries [42]. If this condition prevails and is accentuated with the subsequent expected economic growth, cardiovascular and cardiometabolic epidemics can significantly compromise the well-being of the nation and its public health, making the achieved progress useless. To this day, there are epidemiological differences between Mexico and the developed nations of Europe and North America [43,44]. While in most European nations, CV and coronary incidence and mortality have decreased, in Mexico, they have increased [8]. The median, age-standardized prevalence of HBP varies from 23.8% to 15.7% in middle- and high-income countries. Hypercholesterolemia affects more than 50% of many European nations. Obesity and overweight prevalence have an average value of 53%, while DM affects about 10% of the population.

On the other hand, comparing data with the USA, when the ACC/AHAS cut-off values are used, the rates of HBP are comparable. Obesity and overweight in both countries affect more than four-fifths of the adult populations, and DM is still a little bit higher in Mexico. As can be seen, the epidemiological situation in Mexico is worse. As it happens in Europe, more vascular events occur in the elderly population after many years of negligent diagnosis and insufficient treatment of CV risk factors [43,45,46,47].

As a perspective, trials exploring the TG/HDL ratio and LDL/HDL ratio to determine their role in cardiovascular risks in Mexicans are initiated.

This study has several limitations, such as the inability to measure triglycerides and HDL concentrations and the lack of a record of specific treatments. We believe that the data’s relevance is that, whatever the treatment, only half of the hypertensive subjects are aware of their disease and only half of these are under treatment (any antihypertensives). Only 8% of all subjects with hypertension are under control.

Data showed low treatment compliance and/or efficacy; we do not know who is responsible (patient? physician? economy? medication?). More work is necessary to understand this phenomenon.

## Figures and Tables

**Table 1:** Anthropometric, metabolic, and blood pressure data.

Weight, kg Mean ± SD	BMI, kg/m^2^ Mean ± SD	TC, mg/dL Mean ± SD	Glycemia, mg/dL Mean ± SD	SBP, mm Hg Mean ± SD	DBP, mm Hg Mean ± SD
All, N = 297,370					
72.64 ± 14.28	27.8 ± 4.76	192.5 ± 35.62	95.33 ± 43.81	118.4 ± 16.19	78.17 ± 18.53
Women, n = 161,264					
67.64 ± 13.1	27.99 ± 5.25	193.6 ± 36.2	94.84 ± 45.52	117.1 ± 17.22	77.06 ± 22.91
Men, n = 136,106					
78.56 ± 13.2	27.57 ± 4.05	191.2 ± 34.86	95.91 ± 41.68	119.9 ± 14.74	79.49 ± 11.19

SD, standard deviation; BMI, body mass index; TC, total cholesterol; SBP, systolic blood pressure; DBP, diastolic blood pressure. See text for details.

**Table 2:** Multiple regression equations of total cholesterol, systolic blood pressure, and serum glucose vs. body mass index.

Gender	Risk Factor	Equation
Women	Total cholesterol, mg/dL	TC = 18.21 + 3.936 (BMI) − 0.1087 (age) + 0.7374 (glucose)
	Systolic blood pressure, mm Hg	SBP = 67.64 + 0.4703 (BMI) − 0.1544 (cholesterol) + 0.6989 (glucose)
	Body mass index, kg/m^2^	BMI = −0.4008 + 0.03528 (age) + 0.2141 (cholesterol) − 0.1543 (glucose)
Men	Total cholesterol, mg/dL	TC = −80.70 + 10.13 (BMI) + 0.2935 (age) + 0.2012 (glucose)
	Systolic blood pressure, mm Hg	SBP = 100.8 − 1.593 (BMI) + 0.1072 (cholesterol) + 0.4391 (glucose)
	Body mass index, kg/m^2^	BMI = 9.297 − 0.02189 (age) + 0.0912 (cholesterol) + 0.1612 (glucose)

## Data Availability

Data sharing is available under request.
